# Pruritic rash secondary to cendakimab, an investigative IL-13 inhibitor

**DOI:** 10.1016/j.jdcr.2025.11.029

**Published:** 2025-11-29

**Authors:** Darartu M. Gamada, Jennifer Crimmins, David A. Leiman, Melodi Javid Whitley

**Affiliations:** a School of Medicine, Duke University, Durham, North Carolina; b Department of Pathology, Duke University, Durham, North Carolina; c Department of Gastroenterology, Duke University, Durham, North Carolina; d Department of Dermatology, Duke University, Durham, North Carolina

**Keywords:** cendakimab, drug reaction, eosinophilic esophagitis, IL-13 inhibitor

## Introduction

Cendakimab is an anti-IL-13 monoclonal antibody in development with orphan drug designation through the FDA. The agent has shown potential as a treatment for atopic dermatitis and eosinophilic esophagitis. Therefore, careful monitoring for potential adverse effects will be crucial. A phase 2 trial of cendakimab in atopic dermatitis patients reported adverse effects including COVID-19, upper respiratory infections, and allergic conjunctivitis.^1^ In this report, we present a case of a drug induced erythematous plaque in the gluteal area that resolved with cendakimab cessation.

## Case report

**Fig 1:**
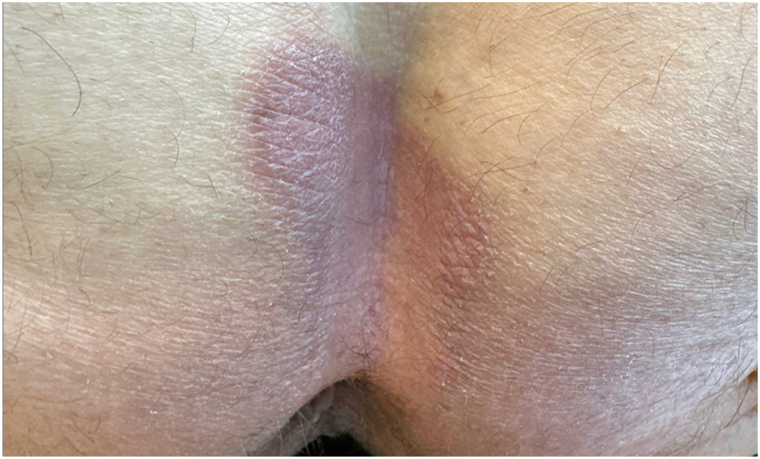
Clinical manifestation: An erythematous plaque was noted in the gluteal area.

A 60-year-old man with hypertension was enrolled in a randomized, double-blind phase 3 trial of cendakimab in patients with eosinophilic esophagitis. He received weekly subcutaneous injections of cendakimab 360 mg per protocol. Five months after starting the medication, he presented to dermatology with 3 months of worsening pruritus of the buttocks, which progressed into a rash spreading to the lower back (Fig 1).

He also reported pruritus of the eyes and ears. Prior treatment for presumed fungal etiology with topical miconazole was ineffective. In dermatology clinic, exam revealed erythematous plaques with lichenified borders on the medial bilateral buttocks and hyperpigmented patches on the lower back. Lichen simplex chronicus was initially favored, and the patient was started on tacrolimus 0.03 % ointment for his buttocks and eyelids, but it was used only once. Additional doses of cendakimab were held at that time. The patient returned to dermatology clinic at one week and reported improvement of pruritus with persistent rash. Exam was significant for well-defined symmetric erythematous plaques in the gluteal cleft. Differential included mycosis fungoides, inverse psoriasis, tinea, and symmetrical drug-related intertriginous and flexural exanthema (SDRIFE).

**Fig 2:**
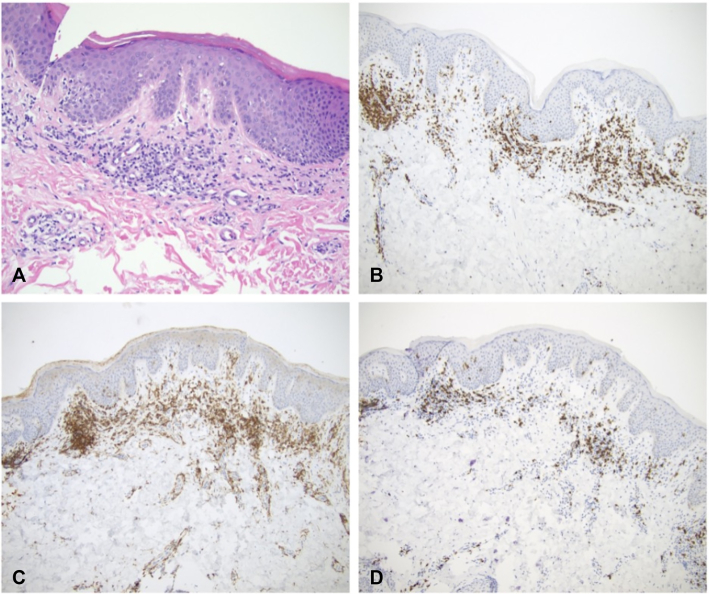
Histopathologic features: H&E sections (**A;** original magnification ×20) demonstrate perivascular and interstitial lymphocytic infiltrate with focal tagging of lymphocytes along the dermal-epidermal junction, which is further highlighted by CD3 immunohistochemical stain (**B;** original magnification ×10). CD4 immunohistochemical stain predominated over CD8 (**C** and **D,** original magnification ×10, respectively).

Punch biopsy revealed a perivascular to interstitial infiltrate composed of lymphocytes and occasional eosinophils. The lymphocytes demonstrated foci of affinity for the epidermis and follicular epithelium, including focal tagging and lining at the dermal-epidermal junction, without significant background spongiosis (Fig 2).

The lymphocytes were highlighted by CD3. CD4 slightly predominated over CD8. CD7 expression was approximately 50%. PASD stain was negative for fungal organisms. T-cell clonality studies for TCR gamma and beta were negative. Two months after cendakimab cessation, only a small light pink lichenified plaque remained on the left medial buttocks. Triamcinolone acetonide 0.1 % topical cream was recommended as treatment for the remaining lesion, and the patient now manages eosinophilic esophagitis with a proton pump inhibitor.

## Discussion

This case details a previously unreported cutaneous drug eruption that temporally aligned with administration of the monoclonal IL-13 antibody, cendakimab. The patient treated the lesion with tacrolimus, which could confound histopathology results. While the patient only used tacrolimus once, the calcineurin inhibitor could have decreased spongiosis, acanthosis, and inflammatory infiltrate.^2^ Given the nonspecific clinical presentation and histopathology findings, a broad differential was considered, including SDRIFE, psoriasis, tinea, and CTCL.

Pathology findings for SDRIFE most commonly include superficial perivascular lymphocytic infiltrate, dermal eosinophils, spongiosis, and orthokeratosis.^3^ While pathology for this case revealed perivascular infiltrate and occasional eosinophils, spongiosis and orthokeratosis were not appreciated. Initial clinical presentation with an erythematous plaque in the gluteal cleft resembled SDRIFE.^4^ However, SDRIFE classically involves a symmetrical lesion of another flexural or intertriginous area, which the patient lacked.

Drug-induced inverse psoriasis was also considered, but pathology lacked characteristic features, such as parakeratosis and elongation of rete ridges. Tinea had initially been on the differential, but treatment with miconazole was ineffective. PASD stain was also negative for fungal microorganisms, so tinea was considered unlikely.

In this case, patient medication history raised particular concern for CTCL. A retrospective cohort study reported increased CTCL risk in patients taking dupilumab, a monoclonal antibody inhibiting both IL-4 and IL-13.^5^ Case reports have also described patients that experienced worsening of CTCL following dupilumab initiation.^6^ Given the overlapping mechanisms of cendakimab and dupilumab targeting IL-13, concern arose while evaluating this patient that cendakimab could similarly be associated with CTCL. On biopsy, perivascular lymphocytic infiltrate, lymphocytes demonstrating foci of affinity for the epidermis, and absence of spongiosis were consistent with CTCL.^7^ However, negative TCR Gamma and Beta studies ruled out CTCL. The patient ultimately had resolution of the erythematous plaque in their gluteal area following cessation of cendakimab, as would be expected for a drug reaction.

While cendakimab has shown promise in treating eosinophilic esophagitis and atopic dermatitis, it is a relatively new agent with limited data on adverse effects. This report describes a case of a novel drug eruption secondary to cendakimab and highlights the difficulty of diagnosis due to overlapping features with SDRIFE, CTCL, psoriasis, and fungal infections.

## Conflicts of interest

None disclosed.
